# Author Correction: Reduced ADP off-rate by the yeast CCT2 double mutation T394P/R510H which causes Leber congenital amaurosis in humans

**DOI:** 10.1038/s42003-023-05357-1

**Published:** 2023-09-20

**Authors:** Mousam Roy, Rachel C. Fleisher, Alexander I. Alexandrov, Amnon Horovitz

**Affiliations:** Department of Chemical and Structural Biology, Weizmann Institute of Science, Rehovot, 7610001 Israel

**Keywords:** Chaperones, Chaperones

Correction to: *Communications Biology* 10.1038/s42003-023-05261-8, published online 29 August 2023.

The original version of the Article contained errors in the reference list.

The journal name in reference 24 has now been corrected to *Invest. Ophthalmol. Vis. Sci*.

Minor errors have also been corrected in references 3, 10 and 19. The original article has been corrected.

