# Supplementary material for: Reduced ADP off-rate by the yeast CCT2 double mutation T394P/R510H which causes Leber congenital amaurosis in humans
Source: Commun Biol. 2023 Aug 29;6:888. doi: 10.1038/s42003-023-05261-8 (PMC10465592; doi:10.1038/s42003-023-05261-8)
Supplement: Supplementary file 2 — Supplementary Information [file 42003_2023_5261_MOESM2_ESM.pdf]

Supplementary Information

Reduced ADP off-rate by the yeast CCT2 double mutation T394P/R510H which causes Leber congenital amaurosis in humans

Mousam Roy[1], Rachel C. Fleisher[1], Alexander I. Alexandrov and Amnon Horovitz

Dept. of Chemical and Structural Biology, Weizmann Institute of Science, Rehovot 761001, Israel

Correspondence: Amnon.Horovitz@weizmann.ac.il

[1]These authors contributed equally to this work

Contents: Supplementary Figures 1-5, Supplementary Note 1 and Supplementary References

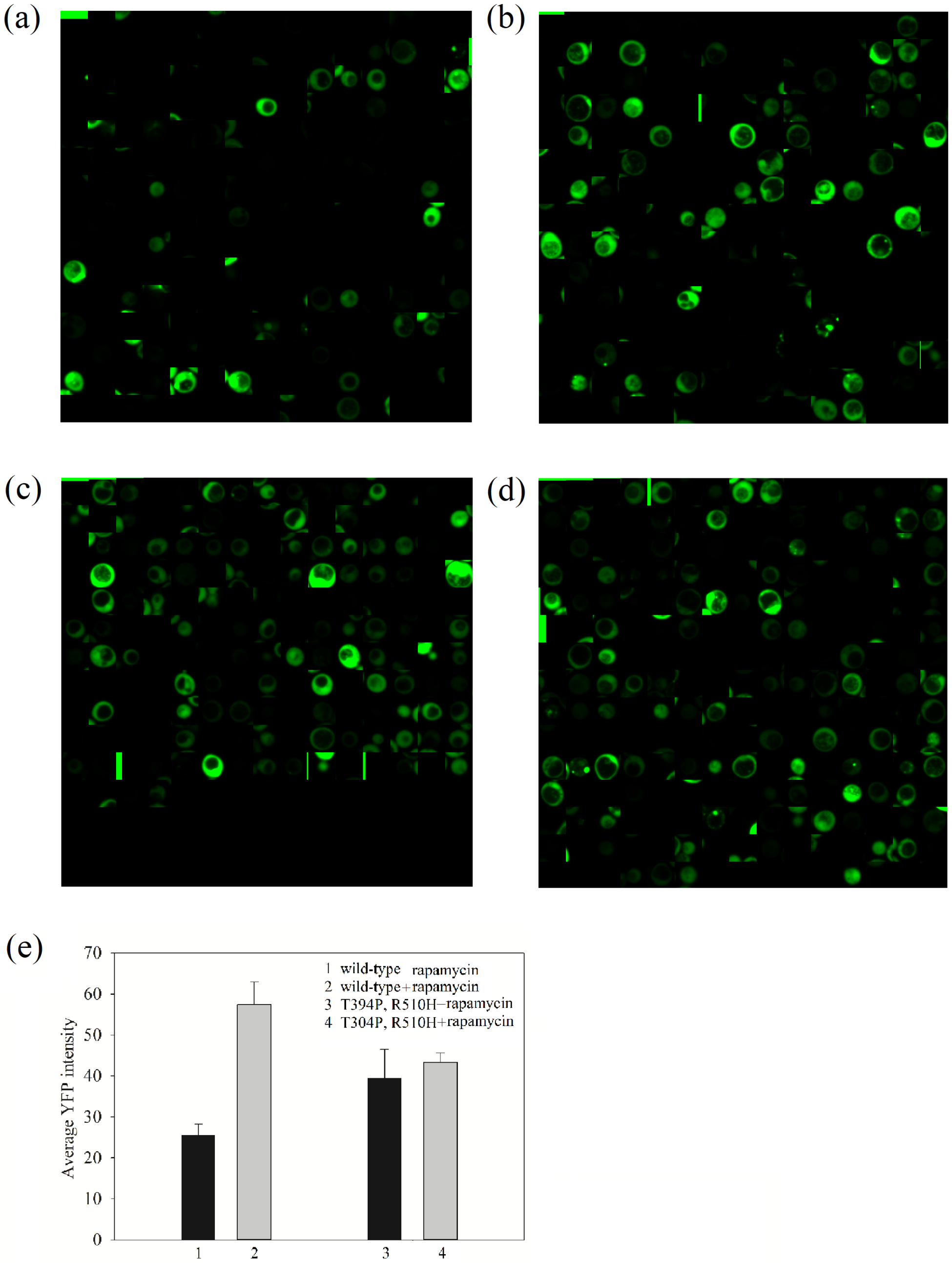

Supplementary Figure 1. The double mutation T394P, R510H in CCT2 impairs autophagy in yeast. Cells containing wild-type CCT/TRiC or the double mutant that express RNQ1-YFP were subjected to rapamycin treatment to induce autophagy. The extent of autophagy was assessed by measuring the fluorescence intensity of YFP inside the vacuoles using ImageJ. Shown are

representative images of cells containing wild type CCT/TRiC with (a) or without (b) rapamycin treatment and of cells containing the double mutant also with (c) or without (d) such treatment. A one-way ANOVA statistical analysis was performed for three independent triplicates using 40-60 images for each condition the averages (+/- standard errors) of which are shown in the bar plot (e). The analysis shows that the increase in YFP intensity in the vacuoles upon rapamycin treatment is significant in the case of cells containing wild-type CCT/TRIC (p-value = 0.007) but not the double mutant (p-value = 0.77), thereby indicating that autophagy is impaired in cells with the double mutant.

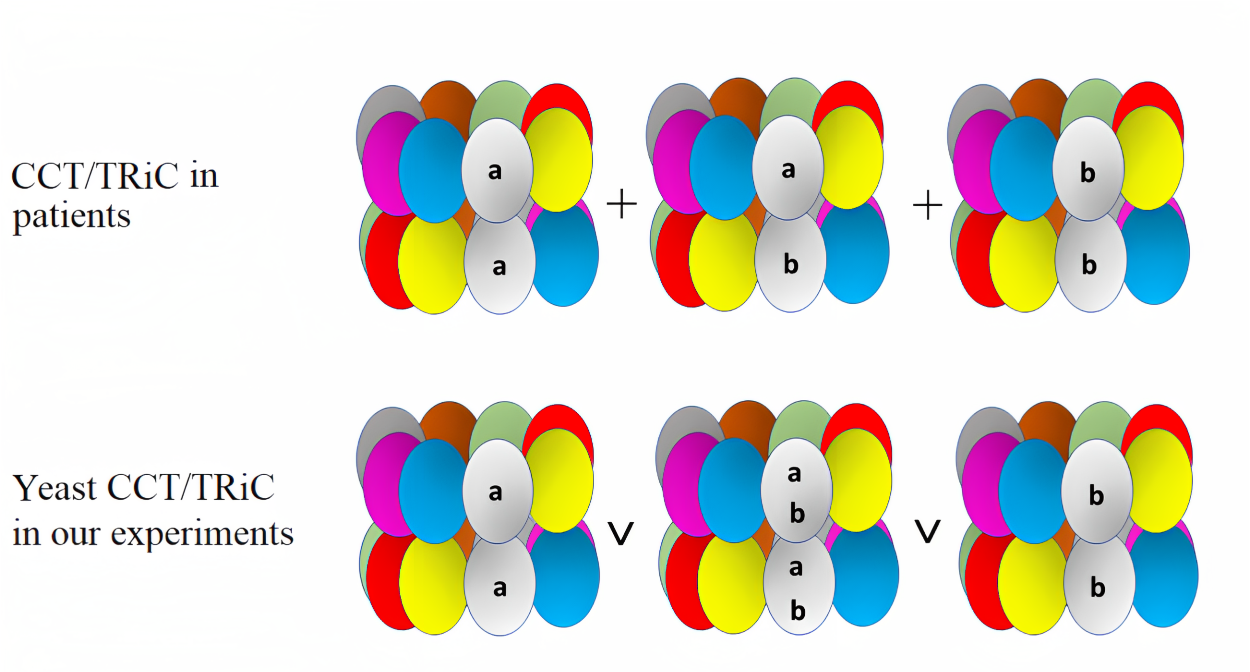

Supplementary Figure 2. Scheme showing CCT/TRiC mutant variants in LCA patients and in this study. The eight different subunits are colored differently in accordance with their order in the ring and the relative orientation of the two rings. Subunit CCT2 is in white. The mutations T400P and R516H in human CCT2 (and the corresponding mutations T394P and R510H in yeast) are designated by a and b, respectively. Patients are heterozygous and, therefore, have three co-existing CCT/TRiC variants with either (i) CCT2(T400P) in both rings (aa); (ii) CCT2(R516H) in both rings (bb); or (iii) CCT2(T400P) in one ring and CCT2(R516H) in the other ring (ab). The aa, ab, and bb variants will be formed at a 1:2:1 binomial ratio if the two different mutant subunits are expressed equally and inserted independently of each other into the two rings. In our study in yeast, the cells contain either one of the two single mutants (which correspond to the aa and bb variants) or the double mutant in which both rings contain CCT2 with the two mutations.

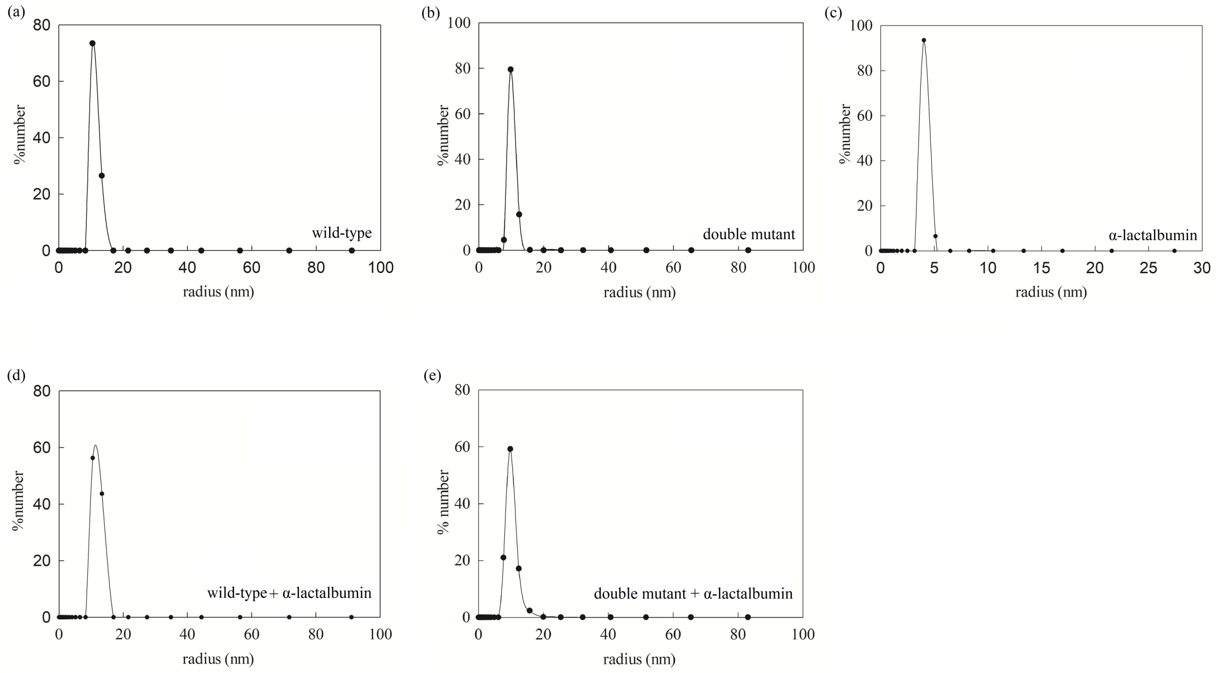

Supplementary Figure 3. Dynamic light scattering measurements of wild-type CCT/TRiC and the T394P, R510H double mutant in the presence and absence of α-lactalbumin. Both wild-type CCT/TRiC (a) and the T394P, R510H double mutant (b) at concentrations of 2.6 μM are found to have narrow size distributions that indicate that more than 99% of the molecules have an apparent hydrodynamic radius of 11-12 nm, which is consistent with CCT/TRiC's structure. By contrast, α-lactalbumin at a concentration of 33 μM forms larger polydisperse aggregates (c). These aggregates are absent when 16.5 μM α-lactalbumin is mixed with 1.3 μM wild-type CCT/TRiC (d) or the T394P, R510H double mutant (e), thereby indicating α-lactalbumin binding. The size distributions of wild-type CCT/TRiC and the T394P, R510H double mutant are not affected in the presence of α-lactalbumin, thereby indicating that α-lactalbumin binding to CCT/TRiC is not destabilizing.

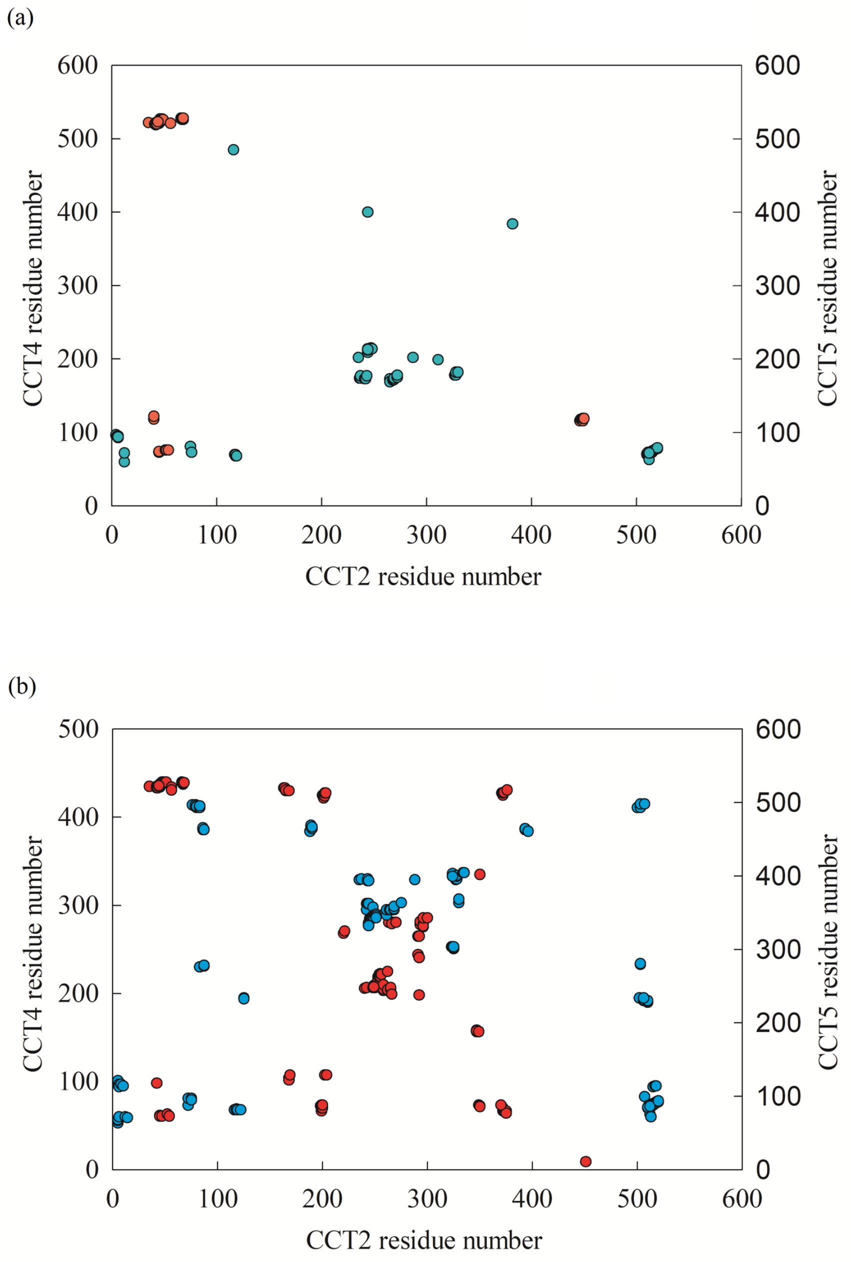

Supplementary Figure 4. Contact maps of subunit CCT2 with its intra-ring neighboring subunits CCT4 and CCT5 in the open (a) and closed (b) states of CCT/TRiC. Contact maps for the open (PDB ID: 5GW4) and closed (PDB ID: 4V8R) states of CCT/TRiC were generated with PDBsum. A dot indicates that residue i in CCT2 and residue j in CCT4 (red) or CCT5 (cyan) are in contact. Two residues are defined as being in contact if at least distance between two of their respective heavy atoms is ≤ 4 Å.

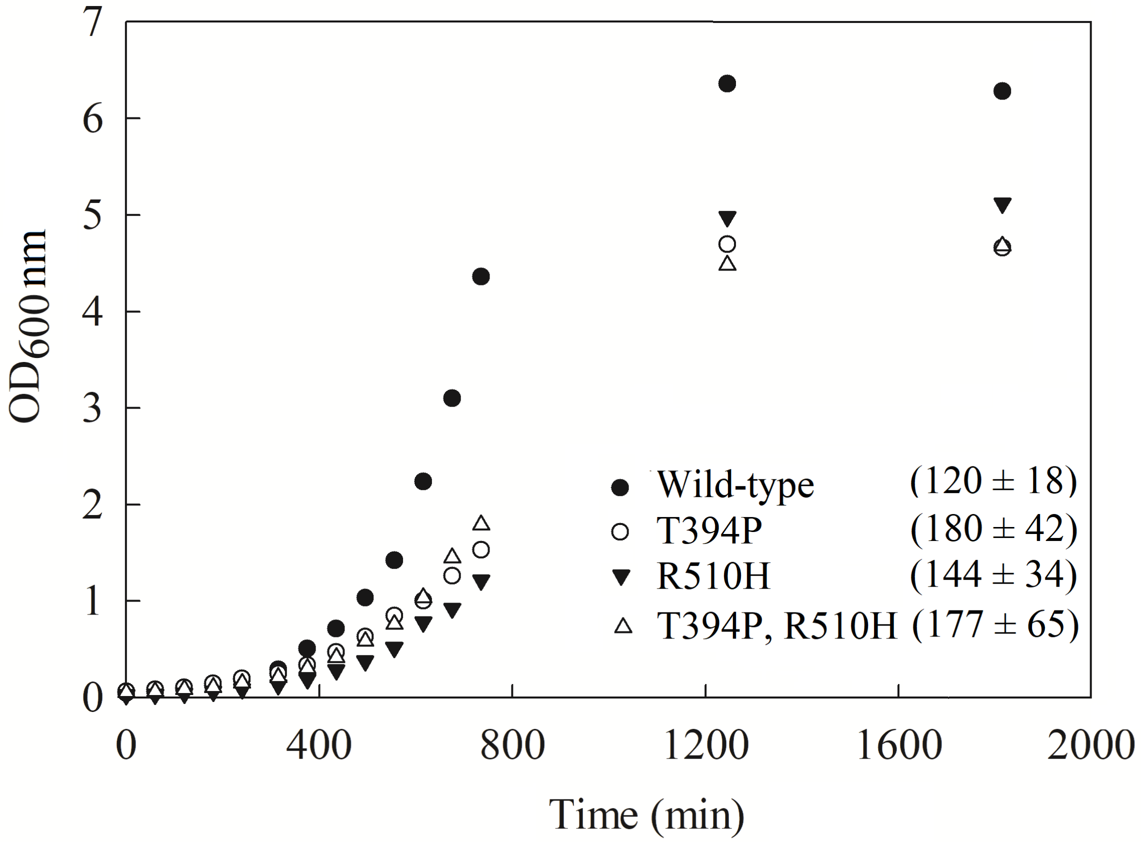

Supplementary Figure 5. Growth curves of haploid *S. cerevisiae* yeast cells containing the T394P and R510H single mutations and the corresponding double mutant in subunit CCT2. 5 ml cell cultures were grown overnight in YPD medium containing 300 μg/ml geneticin at 30 °C and then diluted to an initial $OD_{600\ nm}$ of 0.05. Growth was then continued at 30 °C and the $OD_{600\ nm}$ was measured at different times. The doubling times (± standard deviations) in minutes of the different strains are shown in the inset.

Supplementary Note 1

The analysis of the time course of ATP hydrolysis by the CCT/TRiC T394P, R510H double mutant is based on the scheme in Fig. 4. In this scheme, T and R designate low and high affinity states of apo CCT/TRiC, respectively, T_aX_m and T_bX_n, designate the T state with m and n molecules of X (X = ATP or ADP) bound to different groups, a and b, of subunits and $RATP_q$ designates the R state with q molecules of bound ATP. According to this scheme, the rates of changes in the concentrations of T, T_aS_m and R_aS_m are given by:

$$d[T_a]/dt = -k_1[T_a]\,[ATP]^m + k_{-1}[T_aATP_m] \quad (S1)$$

$$d[T_aATP_m]/dt = k_1[T_a]\,[ATP]^m - (k_{-1} + k_{cat1})[T_aS_m] \quad (S2)$$

$$d[T_aADP_m]/dt = k_{kcat1}\,[T_aATP_m] - (k_3)[T_aADP_m] \quad (S3)$$

Assuming that the value of k_3 is relatively small and can be neglected, two rate constants, τ_1 and τ_2, are obtained for Eqs. (S1) to (S3) that can be expressed, as follows:

$$\tau_1 + \tau_2 = k_1[ATP]^m + k_{-1} + k_{cat1} \quad (S4)$$

$$\tau_1\tau_2 = k_1k_{cat1}[ATP]^m \quad (S5)$$

Assuming that ATP binding and dissociation take place faster than the catalytic step (i.e. $\tau_1 = k_1[ATP]^m + k_{-1}$), one obtains:

$$\tau_2 = k_1k_{cat1}[ATP]^m/(k_1k_{cat1}[ATP]^m + k_{-1}) = k_{cat1}[ATP]^m/([ATP]^m + K_{d1}) \quad (S6)$$

where K_{d1} is the ATP dissociation constant of the 'a' sites. Given that $[T_aADP_m] = A_1(1\text{-}\exp(-\tau_2 t))$ where A_1 is a constant, it follows that the amount of inorganic phosphate (P_i) that is generated by T_aATP_m is given by:

$$[P_i]\{T_aATP_m\} = A_1(1 - \exp(-(k_{cat1}[ATP]^m/([ATP]^m + K_{d1}))t)) \quad (S7)$$

Inspection of Eq. (S7) shows that the rate of P_i production is expected to increase with increasing ATP concentration in a hyperbolic fashion.

Similar considerations to the ones above lead to an analogous expression for the amount of phosphate that is generated by T_bATP_n:

$[P_i]\{T_bATP_n\} = A_2(1- \exp(-(k_{cat2}[ATP]^n/([ATP]^n + K_{d2}))t))$ (S8)

where A2 is a constant and K_{d2} is the ATP dissociation constant of the b sites. Here, it was assumed, as before, that the value of k_{cat2} is relatively small and can be neglected and that ATP binding and dissociation take place faster than the catalytic step.

A third source of P_i is ATP hydrolysis by R, the rate of which is:

$d[P_i]/dt\{R\}= k_{cat3}[RATP_q]$ (S9)

The rate of change in the concentration of $[RATP_q]$ is given by:

$d[RATP_q]/dt = k_5[ATP]^q[R] - (k_{-5} + k_{cat3})[RATP_q]$ (S10)

Assuming that the total concentration of CCT/TRiC, $[E]_T$, can be expressed as $[E]_T \approx [R] + [RATP_q]$ (because by the time that $[RATP_q]$ becomes significant the concentrations of the other species is low), one can rewrite Eq. (S10) as follows:

$d[RATP_q]/dt = k_5[ATP]^q[E]_T - (k_{-5} + k_5[ATP]^q + k_{cat3})[RATP_q]$ (S11)

Integration of Eq. (S11) yields:

$[RATP_q] = (k_5[ATP]^q[E]_T/(k_{-5} + k_5[ATP]^q + k_{cat3}))(1 - \exp(-(k_{-5} + k_5[ATP]^q + k_{cat3})t))$ (S12)

Combining Eqs. (S9) and (S12) and integration, therefore, yields:

$[P_i]\{R\}= k_{cat3}(\alpha/\beta)t + k_{cat3}(\alpha/\beta^2)(\exp^{-\beta t} - 1)$ (S13)

where $\alpha = k_5[ATP]^q[E]_T$ and $\beta = k_{-5} + k_5[ATP]^q + k_{cat3}$. It may be seen that the value of the slope of the linear term in Eq. (S13) has a hyperbolic dependence on ATP concentration, which is consistent with this term corresponding to the steady-state phase of activity.

The total amount of P_i produced is given by combining Eqs. (S7), (S8) and (S13). The resulting equation has the form of:

$$[P_i] = A_1(1- \exp(-k_{obs(1)}t)) + A_2(1- \exp(-k_{obs(2)}t)) + A_3(\exp(-k_{obs(3)}t) - 1)+Vt \quad \text{(S14)}$$

where A_i and $k_{obs(i)}$ are the amplitudes and apparent rate constants, respectively, and V is the steady-state reaction velocity. Eq. (S14) was used to fit the data of the amount of P_i generated by the CCT/TRiC mutant as a function of time.

Eq. (S14) also describes the transient kinetics of ATP hydrolysis of wild-type CCT/TRiC.[1] In the case of wild-type CCT/TRiC, however, the values of $k_{obs(1)}$ and $k_{obs(2)}$ decrease with increasing ATP concentrations in accordance with a conformational selection mechanism. The derivation of Eq. (A8) in ref. 1 for the dependence of $k_{obs(1)}$ and $k_{obs(2)}$ on ATP concentration, in the case of conformational selection, can be modified by (i) not neglecting the catalytic rate constants associated with the burst phases and (ii) recognizing that the amount of inorganic phosphate (P) produced in the burst phase is proportional to the concentration of ATP-bound CCT/TRiC complex (T_1S_m and T_3S_n in Fig. 5 of ref. 1) formed in this phase. Eq. (A8) and subsequent equations in ref. 1 remain unchanged except that K_d becomes K_m.

The effect of the slower ADP off-rate on the amount of free CCT2 can be estimated from the fraction of CCT2 in the free form in wild-type CCT/TRiC (f_{wt}) relative to the mutant (f_{mut}):

$$f_{wt} = [M]/(2[C] + 2[O] + [M]) = K_1K_2/(2 + 2K_1 + K_1K_2) \quad \text{(S15)}$$

$$f_{mut} = [M]/(2[C] + 2[O] + [M]) = 0.5K_1K_2/(2 + K_1 + 0.5K_1K_2) \quad \text{(S16)}$$

where M, O and C designate free CCT2 and the open and closed forms of the CCT/TRiC complex and $K_1 = [O]/[C]$ and $K_2 = [M]/[O]$. It is easy to see by combining Eqs. (S15) and (S16) that $f_{mut}/f_{wt} \approx 0.5$.

Supplementary References

1. Korobko, I., Nadler-Holly, M. & Horovitz, A. Transient kinetic analysis of ATP hydrolysis by the CCT/TRiC chaperonin. *J. Mol. Biol.* **428**, 4520-4527 (2016).
